# Cerebrospinal Fluid MicroRNAs as Early Biomarker Candidates for Predicting Vasospasm Following Aneurysmal Subarachnoid Hemorrhage

**DOI:** 10.3390/genes16091025

**Published:** 2025-08-29

**Authors:** Emre Ozkara, Ozlem Aykac, Ebru Erzurumluoglu Gokalp, Nazli Durmaz Celik, Sara Khadem Ansari, Zehra Uysal Kocabas, Ertugrul Colak, Sinem Kocagil, Zuhtu Ozbek, Oguz Cilingir, Ali Arslantas, Atilla Ozcan Ozdemir, Sevilhan Artan

**Affiliations:** 1 Department of Neurosurgery, Faculty of Medicine, Eskisehir Osmangazi University, Eskisehir 26060, Turkey; dremreozkara@gmail.com (E.O.); zuhtuozbek@gmail.com (Z.O.); draliarslantas@gmail.com (A.A.); 2 Department of Neurology, Faculty of Medicine, Eskisehir Osmangazi University, Eskisehir 26060, Turkey; drzlm@yahoo.com (O.A.); drzuysal@hotmail.com (Z.U.K.); atillaozcanozdemir@gmail.com (A.O.O.); 3 Department of Medical Genetics, Faculty of Medicine, Eskisehir Osmangazi University, Eskisehir 26060, Turkey; ebruerzurumluoglu@gmail.com (E.E.G.); sara.ansari1989@gmail.com (S.K.A.); sinemkocagil@gmail.com (S.K.); drozi1@gmail.com (O.C.); sartan26@gmail.com (S.A.); 4 Department of Biostatistics, Faculty of Medicine, Eskisehir Osmangazi University, Eskisehir 26060, Turkey; ecolak@ogu.edu.tr

**Keywords:** microRNA, cerebrospinal fluid, vasospasm, subarachnoid hemorrhage, biomarker, prognosis

## Abstract

**Background/Objectives**: Aneurysmal subarachnoid hemorrhage (aSAH) is frequently complicated by cerebral vasospasm, a major contributor to delayed cerebral ischemia and poor neurological outcomes. Early prediction remains challenging, and there is a critical need for reliable biomarkers. MicroRNAs (miRNAs) in cerebrospinal fluid (CSF) have emerged as promising indicators of acute neuropathological changes. This study aimed to evaluate CSF miRNA expression profiles in patients with aSAH to identify early predictors of vasospasm and improve clinical risk stratification. **Methods**: We conducted a prospective observational study involving 48 patients (40 patients with aSAH (20 who developed vasospasm, 20 who did not) and 8 healthy controls). A panel of 20 candidate miRNAs was analyzed in CSF samples collected on days 1 and 5 post−hemorrhage using quantitative real−time PCR. Expression differences between groups were assessed, and receiver operating characteristic (ROC) curves were used to evaluate diagnostic performance. **Results**: Several miRNAs were differentially expressed in patients who developed vasospasm. On day 1, miR−221−3p and miR−183−5p were significantly upregulated (*p* = 0.014, *p* = 0.009), while miR−126, miR−29a, and miR−27b−3p were downregulated (*p* = 0.006, 0.021, 0.028) compared with controls. MiR−126 remained suppressed on day 5 (*p* = 0.002). These early changes showed high predictive accuracy (e.g., day 1 AUC for miR−221−3p ≈ 0.98, 95% CI 0.83–1.00). Compared with non−vasospasm patients, miR−221−3p was lower (0.12−fold), while miR−9−3p and miR−183−5p were higher (13.4−fold and 2.7−fold, respectively; all *p* < 0.01). MiR−24 and miR−21−5p correlated with more severe grades and poorer outcomes (*p* < 0.05). **Conclusions**: Specific CSF miRNAs—particularly miR−221−3p, miR−9−3p, and miR−183−5p—may serve as early biomarkers for vasospasm, warranting further validation in larger cohorts.

## 1. Introduction

Aneurysmal subarachnoid hemorrhage (aSAH) is a life−threatening form of hemorrhagic stroke that, while accounting for only about 5% of all strokes, disproportionately affects relatively young patients (over half are younger than 60 years) [1]. It remains highly lethal; approximately 30% of aSAH patients die during the initial hospitalization, and many survivors suffer permanent neurological disabilities [2]. Poor outcomes result not only from the initial bleeding event but also from various acute and delayed complications such as cerebral ischemia (often due to vasospasm), hydrocephalus, and re−bleeding [3]. Among these, delayed cerebral vasospasm (DCV) is one of the most dreaded complications. DCV has long been recognized as a major contributor to morbidity after aSAH, occurring in roughly one−third of patients within 3–10 days of the aneurysm rupture [4,5]. This prolonged constriction of cerebral arteries can lead to delayed cerebral ischemia (DCI), which is strongly associated with neurologic deterioration and poor long−term outcome [5]. The pathophysiology underlying vasospasm is complex and not fully understood; proposed mechanisms include calcium−mediated vasoconstriction, oxidative stress, endothelial dysfunction, inflammation, apoptosis, and other pathways of secondary brain injury [6]. Clinically, predicting which aSAH patients will develop vasospasm or DCI remains a significant challenge. Currently, management relies on vigilant neurological monitoring and prophylactic measures (such as hemodynamic augmentation and calcium−channel blockade) applied broadly to at−risk patients [5]. To date, no reliable biomarker or scoring system exists to identify those at highest risk for vasospasm before clinical symptoms manifest. This gap underscores a critical need for early predictors of vasospasm that could facilitate proactive interventions and enhance outcomes in aneurysmal subarachnoid hemorrhage (aSAH).

In recent years, microRNAs (miRNAs) have emerged as promising candidates for such biomarkers in neurological diseases. MiRNAs are small (~22−nucleotide) non−coding RNAs that regulate gene expression at the post−transcriptional level [7,8]. They play critical roles in virtually all biological processes, and dysregulation of miRNAs has been implicated in numerous CNS disorders, including stroke and traumatic brain injury [9]. Notably, mounting evidence suggests that miRNAs are involved in the pathophysiology of aSAH and its complications. Several studies have documented changes in miRNA expression in cerebral tissues and blood vessels after aSAH, particularly in the context of vasospasm [9,10]. A key feature that makes miRNAs attractive as clinical biomarkers is their stability in biofluids. MiRNAs circulate in extracellular fluids such as plasma and cerebrospinal fluid (CSF) packaged in exosomes or protein complexes, which protect them from RNase degradation [11,12]. These characteristics—disease association, biological relevance, and stability in CSF/blood—position miRNAs as potential early indicators of vasospasm risk in aSAH.

Recent evidence strongly suggests that cerebrospinal fluid (CSF) microRNAs (miRNAs) are promising candidates for predicting vasospasm following aneurysmal subarachnoid hemorrhage (aSAH). A few studies have also described miRNA expression in CSF, showing different patterns related to vasospasm and DCI on different days after aSAH [10,13,14,15], which can reflect ongoing acute pathophysiological changes in the brain. The sorts of pathways in inflammation, endothelial integrity, and cerebrovascular tone that these miRNAs seem to modify are biologically as well as clinically significant. Given the tissue−specific expression patterns of many miRNAs, aSAH−induced changes in brain−specific miRNAs may provide insight into disease mechanisms. Evaluating miRNAs as biomarkers in neurological conditions, particularly by analyzing their levels in CSF, is, therefore, a promising approach to clarify aSAH pathophysiology and its complications. Studies examining CSF miRNA profiles in aSAH are still minimal; to the best of our knowledge, only a few investigations have focused on miRNA changes in CSF after aSAH.

Nevertheless, the data currently available remain limited by small sample sizes and heterogeneous results, and no single miRNA panel has been established for clinical practice.

To fill this gap, the present study aimed to characterize the expression profiles of a panel of 20 candidate miRNAs in the CSF of aSAH patients and to determine their association with the development of vasospasm and with patient outcomes. By generating initial data on these potential biomarkers, we sought to address a gap in the literature and lay the groundwork for improved prognostic and therapeutic strategies in aSAH.

## 2. Materials and Methods

All procedures were conducted according to the Declaration of Helsinki and approved by the Clinical Research Ethics Committee of Eskisehir Osmangazi University. Written informed consent was obtained from all participants. The well−established methods used are briefly described and appropriately cited below.

### 2.1. Study Design and Participants

This prospective study was conducted at the neurosurgery clinic of our university hospital. All aSAH patients were managed by an experienced neurovascular team, and all molecular analyses were performed in a blinded fashion in the medical genetics department. Clinical data were not revealed to the genetics researchers until after miRNA expression results were obtained. The study cohort consisted of 48 individuals: 40 patients with aSAH (20 who developed vasospasm and 20 who did not) and 8 non−SAH control subjects. For each aSAH patient, CSF samples were collected at two time points (on day 1 and day 5 after hemorrhage), yielding a total of 80 patient CSF samples. An additional single CSF sample was obtained from each control, bringing the total number of CSF samples analyzed to 88. In subsequent descriptions, patients with aSAH who developed vasospasm are referred to as the vasospasm−positive group (VSP+), and those who did not develop vasospasm are referred to as the vasospasm−negative group (VSP−).

### 2.2. Inclusion and Exclusion Criteria

A CONSORT−style flow diagram illustrating the patient selection process and group allocation is provided. The inclusion and exclusion criteria for patient and control selection are outlined.

#### 2.2.1. Patient Inclusion Criteria

- Age 18–80 years with acute aSAH confirmed by sudden onset of symptoms (e.g., severe headache, nausea/vomiting, altered consciousness, or focal neurological deficits) and evidence of subarachnoid hemorrhage on cranial computed tomography.
- Identification of a ruptured saccular intracranial aneurysm as the bleeding source by digital subtraction angiography or CT angiography.
- Definitive treatment of the ruptured aneurysm (endovascular coiling or microsurgical clipping) within 48 h of diagnosis.
- Radiological evidence of cerebral vasospasm on CT angiography during the post—hemorrhage course.
- In patients with clinical signs of vasospasm, confirmation of vasospasm by diagnostic cerebral angiography and administration of intra−arterial vasospasm therapy.

#### 2.2.2. Patient Exclusion Criteria

- Treatment for an unruptured aneurysm (i.e., cases without acute hemorrhage).
- History of previous aSAH.
- Family history of intracranial aneurysm or aSAH in first−degree relatives
- Known hereditary conditions predisposing to aneurysm (e.g., polycystic kidney disease or connective tissue disorders).
- Traumatic, mycotic, or dissecting aneurysms.
- Presence of multiple aneurysms.
- Presentation > 72 h after the ictus (delayed admission beyond the acute phase).
- Comorbid conditions (e.g., active malignancy, chemotherapy, recent trauma) that could disrupt the blood–brain barrier or confound neurological assessment.

#### 2.2.3. Control Group

Control individuals were pre−operative patients undergoing elective spinal anesthesia for non−neurological surgeries such as lumbar disc herniation repair. They had no history of subarachnoid hemorrhage, intracranial aneurysm, neurological disease, or chronic systemic illness. Neurological examinations were normal, and no neuroimaging was performed unless clinically indicated. CSF samples (3–4 cc) were obtained prior to surgical intervention. This definition of “clinically healthy volunteer” specifically refers to individuals without known cerebrovascular, neurological, or systemic conditions that might influence CSF composition.

Although the control group consisted of only eight individuals, this limitation was due to ethical and practical challenges in obtaining cerebrospinal fluid (CSF) from truly healthy volunteers. This approach aligns with prior CSF miRNA studies in aSAH using similarly sized control cohorts. Despite the small sample size, the dispersion of ΔCt values among controls was minimal, and statistical differences between groups were robust. To account for the limited sample size, non−parametric tests (Mann–Whitney U tests) were applied. Additionally, individual control values and corresponding variability measures (means ± SDs or medians with IQRs) are provided in scatter plots to ensure transparency.

### 2.3. Clinical Management

The management of subarachnoid hemorrhage patients in our 24 h accredited stroke center clinic was performed by a neurovascular team consisting of specialists in neurosurgery, interventional neurology, and neurological intensive care. The same team also performed postoperative intensive care of all patients. All patients consulted from the emergency department underwent computerized brain tomography at the time of initial assessment, followed by emergency brain tomography angiography or diagnostic cerebral angiography, depending on the patient’s clinical condition. Mannitol, furosemide, hyperosmolar treatment, hypertensive agents, nimodipine, and antiepileptic agents were administered when necessary. External ventricular drainage (EVD) was performed based on the patient’s clinical status and the severity of the bleeding. Lumbar drainage (LD) was placed in all surgical patients, and, in those treated endovascularly, if EVD or LD was not placed, CSF samples were obtained by puncture. All patients underwent aneurysm closure as soon as possible, within 48 h at the latest. Tomography angiography was performed on the fifth day for both aneurysm control and vasospasm detection. In patients with suspected vasospasm, diagnostic angiography was performed to confirm the diagnosis. CSF samples were obtained under sterile conditions during routine lumbar drainage (LD), external ventricular drainage (EVD), or via lumbar puncture in patients not requiring drainage. In control subjects, CSF was collected at the time of spinal anesthesia prior to elective surgery. All samples were handled identically, centrifuged immediately, and frozen at −80 °C within 30 min. Both Hunt–Hess and WFNS grading scales were used as they offer complementary clinical insights. Hunt–Hess focuses on neurological symptoms, while WFNS incorporates GCS and motor findings. Including both allowed for more comprehensive severity stratification.

### 2.4. Selection of Target miRNAs

Preliminary selection of candidate miRNAs was based on a focused review of the literature and bioinformatics databases (PubMed, miRBase), identifying those previously implicated in vasospasm or subarachnoid hemorrhage. This review was conducted by examining relevant articles and databases, including PubMed, Embase, Ovid, Google Scholar, and miRBase. Based on this research, the expression profiles of 20 miRNAs (miR−29a, miR−126, miR−200a−3p, miR−451a, miR−146a−5p, miR−1297, miR−27b−3p, miR−502−5p, miR−143−3p, miR−145−5p, miR−17−5p, miR−221−3p, miR−21−5p, miR−15a, miR−9−3p, miR−630, miR−24, miR−148b−3p, miR−497, and miR−183−5p) were determined in the CSF samples of the aSAH/vasospasm+ and aSAH/vasospasm− groups and compared with control CSF samples.

### 2.5. RNA Extraction from CSF Samples

The CSF samples from aSAH cases and controls were centrifuged for 5 min at 2000× *g* at +4 °C to remove contaminated blood cells. CSF material was carefully pipetted from the tube without disturbing the blood pellet, and aliquots were stored at −80 °C until RNA isolation and further analysis. Total RNA was extracted using the miRVANA™ PARIS™ Kit (Applied Biosystems, Foster City, CA, USA) according to the manufacturer’s instructions. Among the 20 miRNAs tested, 5 demonstrated significant expression changes. 

### 2.6. Quantitative Real−Time Polymerase Chain Reaction (qRT−PCR)

Following the manufacturer’s guidelines, complementary DNA (cDNA) was synthesized using the miRNA ALL−In−One cDNA Synthesis Kit (ABM, CA). Each reaction mixture contained 2X miRNA cDNA Synthesis SuperMix (10 µL), 2 µL of Enzyme Mix, 2 µL of total RNA, and 8 µL of nuclease−free water, resulting in a total reaction volume of 20 µL. The reverse transcription process involved the following cycle profile: 37 °C for 30 min, 50 °C for 15 min, 85 °C for 5 min, and a hold at 4 °C.

Quantitative real−time PCR (qRT−PCR) reactions were performed on a CFX−96 Real−Time PCR Detection System (BIO−RAD, C1000 Touch Thermal Cycler) using BrightGreen miRNA qPCR Master Mix (5 μL), forward primer (0.35 μL), reverse primer (0.35 μL), cDNA (2 μL), and nuclease−free water (2.3 μL) to make a final volume of 10 μL. The amplification reaction conditions consisted of an initial incubation at 95 °C for 10 min, followed by 40 cycles of denaturation at 95 °C for 10 s, annealing at 60 °C for 20 s, and extension at 72 °C for 30 s. All samples were analyzed in duplicates.

The housekeeping gene U6 was used as a reference gene for normalizing miRNA expression. U6 small nuclear RNA was selected as the endogenous reference control due to its frequent use in CSF miRNA qRT−PCR studies [16] and its recommendation by the assay manufacturer. Post hoc analysis of U6 Ct values across all samples revealed minimal variation (~1 Ct) between groups, confirming stable expression and validating its use in this setting. Although using a single reference gene has known limitations, our cross−check with another abundant and typically stable miRNA (miR−16−5p) yielded consistent expression patterns. We acknowledge the importance of employing multiple reference genes or spike−in controls (e.g., cel−miR−39) for future studies and recommend applying geNorm or NormFinder algorithms to ensure robust normalization.

The primer catalogue numbers are as follows: U6 (control) CAT:MPH00001, hsa−miR−126−3p CAT:MPH01082, hsa−miR−200a−3p CAT:MPH02296, hsa−miR−451a CAT:MPH02756, hsa−miR−27b−3p CAT:MPH02385, hsa−miR−146a−5p CAT:MPH02204, hsa−miR−502−5p CAT:MPH01742, hsa−miR−1297 CAT:MPH01130, hsa−miR−29a−3p CAT:MPH01310, hsa−miR−15a CAT:MPH01189, hsa−miR−148b−3p CAT:MPH02208, hsa−miR−17−5p CAT:MPH02236, hsa−miR−21−5p CAT:MPH02337, hsa−miR−221−3p CAT:MPH02350, hsa−miR−9−3p CAT:MPH03965, hsa−miR−183−5p CAT:MPH02250, hsa−miR−497 CAT:MPH01733, hsa−miR−24 CAT:MPH01294, hsa−miR−143−3p CAT:MPH02196, hsa−miR−630 CAT:MPH01931, and hsa−miR−145−5p CAT:MPH02201 (all applied Biological Materials, ABM).

The cycle threshold (Ct) values of the reference and target miRNA were determined in each sample, and the relative miRNA level was calculated using the 2−ΔΔCt method.

### 2.7. Statistical Analysis

The statistical analysis was conducted using SPSS version 21.0 (IBM Corporation, NY, USA), while GraphPad Prism v.9.5.1 (GraphPad Software) was used to generate the graphs. The Shapiro–Wilk test was employed to evaluate the distribution of the variables. For standard variation variables, comparisons were made using Student’s t−test, while the Mann–Whitney U test was used to determine differences between the two groups. The diagnostic potential of candidate miRNAs as a biomarker was assessed by constructing receiver operating characteristic (ROC) curves for miRNAs with a significant difference in levels and calculating the area under the ROC curve (AUC), specificity, and sensitivity with a 95% confidence interval (95% CI). A value of *p* < 0.05 was considered statistically significant.

## 3. Results

### 3.1. Demographic Characteristics

A total of 40 patients with aSAH (19 females and 21 males) were included in the analysis, along with eight control subjects (four females and four males). The mean age was similar across the three groups (57.8 ± 14.2 years in the VSP+ group, 57.9 ± 12.0 in the VSP− group, and 57.1 ± 9.7 years in controls; *p* = 0.988). At presentation, the aSAH patients had a range of severity scores. In the VSP+ group, 70% of patients were classified as WFNS grades 1–2 and 30% as grades 3–5, while 60% were Hunt–Hess grades 1–2 and 40% were grades 3–5. According to the CT Fisher scale, 45% of the VSP+ patients had Fisher grade 1–3 hemorrhage, and 55% had grade 4. The 3−month functional outcomes (Modified Rankin Scale, mRS) for all aSAH patients showed that 35% achieved good recovery (mRS 1–2) and 65% had significant disability or worse (mRS 3–6). There were no significant differences between the VSP+ and VSP− patient groups in terms of baseline characteristics such as age, sex, history of hypertension, or initial hemorrhage severity score (Fisher grade, Hunt–Hess grade, WFNS grade, and mRS at presentation were comparable; *p* > 0.05 for all; see Table 1).

### 3.2. miRNA Expression Profiles: Vasospasm−Positive vs. Control

The results reveal statistically significant differences in the expression of five miRNAs in the VSP+ patients on the first day after SAH compared with the control group. We observed a marked increase in the expression of miR−221−3p (*p* = 0.014) and miR−183−5p (*p* = 0.009). In contrast, miR−126 (*p* = 0.006), miR−29a (*p* = 0.021), and miR−27b−3p (*p* = 0.028) were significantly downregulated. Moreover, the expression levels of miR−126 remained significantly downregulated on the fifth day after SAH (*p* = 0.002) (Table 2).

**Figure 1:**
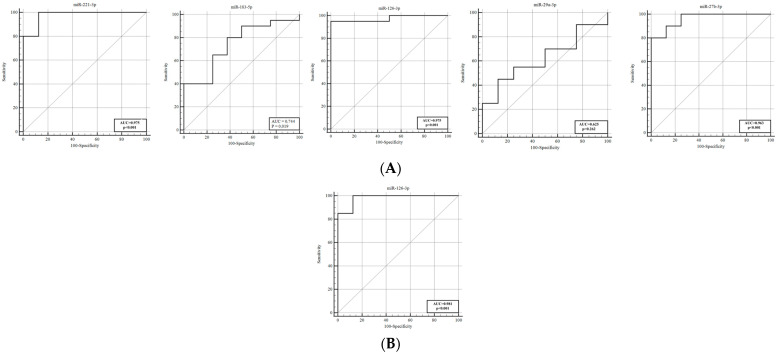
ROC curves to distinguish VSP+ patients from controls. (**A**) The AUCs of miR−221−5p, miR−183−5p, miR−126, miR−29a-3p and miR−27b−3p were 0.975, 0.744, 0.975, 0.625 and 0.963, respectively, on day 1 post−SAH. (**B**) miR−126 achieved an AUC greater than 0.9 on day 5. AUC, area under the curve; ROC, receiver operating characteristic; VSP+, vasospasm−positive group.

ROC analyses were performed to evaluate the diagnostic performance of these 20 miRNAs in distinguishing between VSP+ patients and controls. The ROC analysis indicated that miR−221−3p, miR−126, and miR−27b−3p exhibited high sensitivity and specificity, with AUC values of 0.975, 0.975, and 0.963, respectively (Table 3, Figure 1). Notably, the expression of miR−126 continued to decrease on the fifth day after SAH and demonstrated a significantly high AUC value of 0.981 with 100% sensitivity.

### 3.3. miRNA Expression Profiles: Vasospasm-Negative vs. Control

When comparing the miRNA expression levels on the first day after SAH between VSP (vasospasm−negative) patients and control groups, miR−221−3p was significantly upregulated (31.91−fold). At the same time, miR−9−3p (0.44−fold) and miR−29a (0.26−fold) were downregulated considerably (*p* < 0.001 and *p* = 0.021, respectively). Additionally, a continued downregulation of miR−29a (0.78−fold) was detected on the fifth day after SAH in VSP samples compared with controls (Table 4).

**Figure 2:**
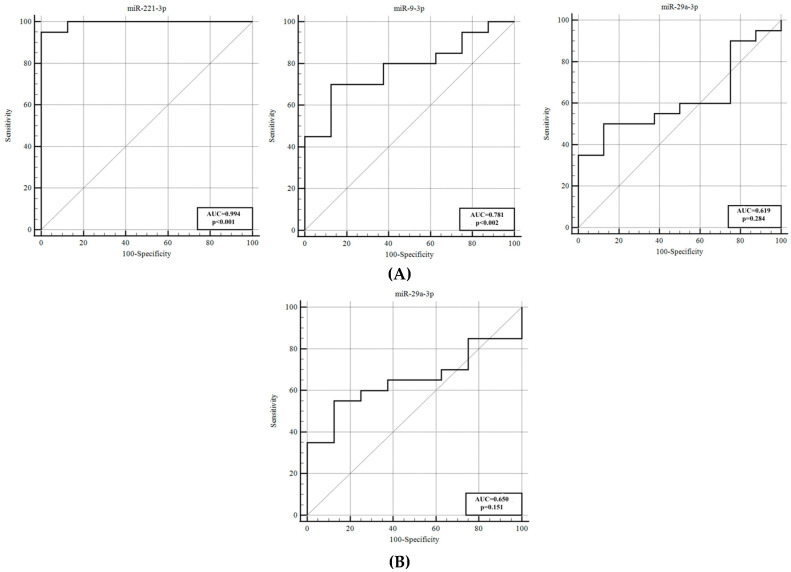
ROC curves of patients with VSP− and controls. (**A**) The area under the ROC curve of miR−221−3p, miR−9−3p and miR−29a was 0.994, 0.781 and 0.619 respectively, on day 1 post−SAH. (**B**) miR−29a had an AUC of 0.650 on day 5. miR−29a was not identified as significant in the ROC curve analysis (*p* = 0.151). AUC, area under the curve; ROC, receiver operating characteristic; VSP−, vasospasm−negative group.

The ROC analysis between these groups indicated that miR−221−3p, with high sensitivity and specificity, exhibited strong discriminative potential, reflected by a high AUC (0.994) value (Figure 2).

### 3.4. miRNA Expression Profiles: Vasospasm vs. No Vasospasm (VSP+ vs. VSP−)

**Figure 3:**
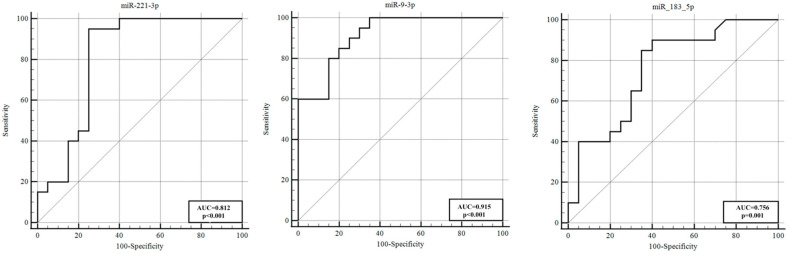
ROC analysis to distinguish VSP+ from VSP− on day 1. The area under the ROC curve of miR−221−3p, miR−9−3p, and miR−183−5p was 0.812, 0.915, and 0.756, respectively. These three miRNAs reached high AUC values, highlighting their role as a potential biomarker panel for early prediction of vasospasm. AUC, area under the curve; ROC, receiver operating characteristic; VSP−, vasospasm−negative group; VSP+, vasospasm−positive group.

A key question was whether CSF miRNA levels before the onset of vasospasm could distinguish those aSAH patients who would develop vasospasm (VSP+) from those who would not (VSP−). We directly compared the VSP+ and VSP− groups at each time point. In the day 1 CSF samples, three miRNAs showed significant differences between the two patient groups (Table 5, Figure 3). MiR−221−3p levels were lower in VSP+ patients on day 1 compared with VSP− patients (a 0.12−fold relative expression, implying an ~8.3−fold decrease; *p* < 0.001). By contrast, miR−9−3p and miR−183−5p were higher in the VSP+ group on day 1 (miR−9−3p was elevated ~13.41−fold (*p* < 0.001) and miR−183−5p ~2.66−fold (*p* = 0.005) in VSP+ vs. VSP−). These early post−SAH differences suggest that, by 24 h, patients fated to develop vasospasm already exhibit a distinct CSF miRNA signature (lower miR−221−3p, higher miR−9−3p/miR−183−5p). In the day 5 samples, no statistically significant differences in miRNA expression were observed between the VSP+ and VSP− groups (after vasospasm had clinically been declared), indicating that the expression differences are most pronounced in the acute phase, before or around the time vasospasm develops. ROC analysis for discriminating VSP+ vs. VSP− on day 1 showed that these three miRNAs had good diagnostic performance. MiR−9−3p was particularly strong, with an AUC of 0.915 (80% sensitivity, 85% specificity; *p* < 0.0001). MiR−221−3p also showed high accuracy (AUC = 0.812, 95% CI 0.658–0.918; 95% sensitivity, 75% specificity; *p* < 0.0001). MiR−183−5p had an AUC of 0.756 (85% sensitivity, 65% specificity; *p* = 0.001). Optimal cut−off values were identified for each (e.g., >−0.89 ΔCt for miR−221−3p; ≤−2.05 ΔCt for miR−9−3p, etc.; see Table 5). These results demonstrate that a panel of miRNAs measured one day after SAH can effectively distinguish patients who will develop vasospasm from those who will not, well before clinical vasospasm manifests. In practical terms, circulating miRNAs, such as miR−221−3p, miR−9−3p, and miR−183−5p, show strong potential as early biomarkers for the risk of vasospasm.

Table 5 shows day 1 CSF miRNAs distinguishing patients with vs. without vasospasm (VSP+ vs. VSP−). Cut−off values are given as ΔCt thresholds. These miRNAs are miR−221−3p (95.0% sensitivity, 75.0% specificity, AUC = 0.812 (95% CI 0.658–0.918), cut−off ΔCt > −0.89, *p* < 0.0001), miR−9−3p (80.0% sensitivity, 85.0% specificity, AUC = 0.915 (95% CI 0.783–0.980), cut−off ΔCt ≤ −2.05, *p* < 0.0001), and miR−183−5p (85.0% sensitivity, 65.0% specificity, AUC = 0.756 (95% CI 0.595–0.878), cut−off ΔCt ≤ 0.86, *p* = 0.001). One day after SAH, these three miRNAs achieved high AUC values and could serve as a biomarker panel to predict vasospasm development with reasonable accuracy. By five days post−SAH, no miRNA showed a significant difference between VSP+ and VSP− groups, suggesting that the window for early prediction is within the first few days.

### 3.5. miRNA Expression and Clinical Severity in Vasospasm Patients

**Figure 4:**
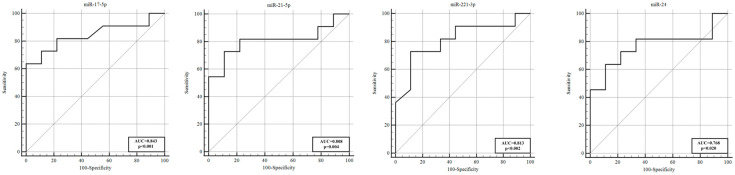
ROC curve analysis for discriminating Fisher grades (1–3) from grade (4) on day 5 post−SAH. The AUCs of miR−17−5p, miR−21−5p, miR−221−3p, and miR−24 were 0.843, 0.808, 0.813, and 0.768, respectively. AUC, area under the curve; ROC, receiver operating characteristic.

**Figure 5:**
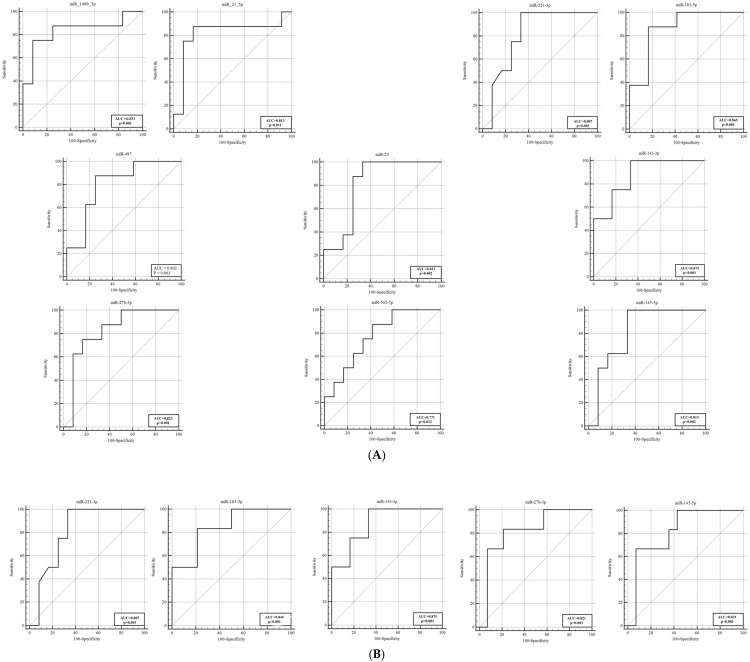
ROC curve analysis for neurological severity. (**A**) miR−148b−3p, miR−21−5p, miR−221−3p, miR−183−5p, miR−497, miR−24, miR−143−3p, miR−27b−3p, miR−502−5p, and miR−145−5p had good predictive value for distinguishing severe Hunt–Hess grades (3–5) from mild (1–2) cases. (**B**) miR−221−3p, miR−183−5p, miR−143−3p, miR−27b−3p, and miR−145−5p achieved AUCs greater than 0.8 in patients with severe WFNS (3–5) vs. mild (1–2). AUC, area under the curve; ROC, receiver operating characteristic.

We further investigated whether miRNA expression in vasospasm patients (VSP+ group) was associated with clinical severity measures, including the Fisher grade (hemorrhage amount on CT), Hunt–Hess and WFNS grades (neurological status), and mRS outcomes. For these analyses, within the VSP+ cohort, we stratified patients by clinical severity and compared miRNA levels between subgroups. By hemorrhage amount (Fisher grade), and within the VSP+ cases, patients with a high Fisher grade (grade 4, indicating diffuse thick SAH) were compared to those with lower Fisher grades (1–3). In the day 5 CSF samples of VSP+ patients, four miRNAs were significantly elevated in the Fisher grade 4 group (severe hemorrhage) compared with grades 1–3: miR−17−5p (~8.10−fold higher, *p* = 0.007), miR−21−5p (~5.88−fold, *p* = 0.020), miR−221−3p (~5.11−fold, *p* = 0.016), and miR−24 (~2.86−fold, *p* = 0.046). All four remained significantly upregulated despite the small subgroup sizes. ROC curve analysis for discriminating high vs. low Fisher grades on day 5 yielded AUCs in the range of ~0.768–0.843 for these miRNAs (miR−17−5p AUC 0.843, miR−21−5p AUC 0.808, miR−221−3p AUC 0.813, miR−24 AUC 0.768; see Table 6, Figure 4). This suggests that, in patients who developed vasospasm, those with a more extensive hemorrhage burden had distinctly higher levels of specific miRNAs by day 5, which could relate to more intense secondary brain injury or inflammation. By neurological grade (Hunt–Hess and WFNS), we categorized VSP+ patients as “mild” or “severe” based on admission neurological scales. For Hunt–Hess, grades 1–2 were considered mild, and grades 3–5 were considered severe. On day 1 post−SAH, miR−148b−3p and miR−21−5p levels were significantly higher in patients with severe Hunt–Hess grades (3–5) compared with those with grades 1–2 (miR−148b−3p 2.80−fold, *p* = 0.012; miR−21−5p 3.14−fold, *p* = 0.025). By day 5, a broader array of miRNAs were elevated in the severe Hunt–Hess group. MiR−221−3p (2.37−fold, *p* = 0.020), miR−183−5p (4.09−fold, *p* = 0.005), miR−497 (2.85−fold, *p* = 0.025), miR−24 (2.51−fold, *p* = 0.020), miR−143−3p (3.93−fold, *p* = 0.004), miR−27b−3p (2.46−fold, *p* = 0.039), miR−502−5p (2.72−fold, *p* = 0.047), and miR−145−5p (2.46−fold, *p* = 0.020) were all significantly higher in severely graded patients on day 5. Thus, higher acute clinical severity was associated with greater increases in multiple miRNAs over the first five days. For WFNS grade (another clinical grading system, with grades 1–2 classified as mild and 3–5 as severe), we observed a similar pattern. In the VSP+ group, miR−221−3p on day 1 was significantly higher in patients with severe WFNS (3–5) vs. mild (1–2) (which is interesting because miR−221−3p was generally lower in VSP+ vs. VSP— overall, but among VSP+ perhaps those with a worse clinical state had a rebound increase—this discrepancy might reflect timing or subgroup factors). On day 5, miR−183−5p, miR−143−3p, miR−145−5p, and miR−27b−3p were significantly higher in the severe WFNS group than in the mild group. The ROC analysis for neurological severity (Table 7, Figure 5) indicated that several of these miRNAs had good predictive value for distinguishing severe vs. mild cases (e.g., miR−148b−3p AUC 0.833, miR−21−5p AUC 0.813 for Hunt–Hess; miR−183−5p AUC 0.845 for WFNS). For example, in Hunt–Hess 3–5 vs. 1–2, miR−183−5p achieved 87.5% sensitivity and 83.3% specificity (AUC 0.865, *p* < 0.001). These data suggest that certain miRNAs (like miR−21−5p, miR−24, miR−143−3p, and miR−183−5p) correlate with the severity of the initial hemorrhage and the degree of neurological impairment in vasospasm patients, potentially serving as prognostic biomarkers for outcome.

### 3.6. Summary of Key miRNA Findings

In summary, our results reveal that aSAH patients who develop vasospasm have a distinct CSF miRNA expression profile in the acute phase, differing from both healthy controls and aSAH patients without vasospasm. The most notable early changes associated with impending vasospasm (within 24 h post−bleed) were an increase in miR−221−3p and miR−183−5p (vs. controls) coupled with a decrease in miR−221−3p (vs. non−vasospasm patients) and an increase in miR−9−3p. These specific miRNAs demonstrated strong diagnostic performance for vasospasm prediction (AUC ~0.8–0.92) one day after SAH, well before clinical vasospasm became evident. By day 5, miR−126 emerged as a sustained differentiator (remaining low in vasospasm patients). We also identified miR−24 as a candidate marker. Although not significantly changed on day 1, miR−24 was significantly elevated by day 5 in the vasospasm group. It was strongly associated with poorer clinical status (higher in patients with severe grades and worse outcomes). In addition, miR−21−5p and miR−221−3p levels on day 5 were higher in patients with poor neurological outcomes, suggesting a possible role in prognostication. Our findings suggest that a multi−miRNA panel—comprising miR−221−3p, miR−9−3p, and miR−183−5p for early detection and miR−21−5p and miR−24 for severity stratification—could serve as the foundation for a predictive model. Rather than relying on a single biomarker or ratio, this panel could be implemented through multivariate logistic regression to estimate vasospasm risk. Similar multi−marker approaches have been used in prior neurovascular studies with improved predictive power. Future validation studies will aim to refine and validate such a panel in larger cohorts.

## 4. Discussion

Cerebral vasospasm (CVS) is a significant complication of aSAH that contributes heavily to delayed cerebral ischemia (DCI), long−term disability, and mortality [17]. The underlying pathological mechanisms of vasospasm are not yet fully understood, but appear to involve a multifactorial cascade including calcium−dependent and −independent vasoconstriction, oxidative stress, endothelial dysfunction, inflammation, apoptosis, autophagy, and altered gene expression [9,18,19]. Early identification of patients at high risk for vasospasm—before clinical symptoms manifest—is crucial for preventing secondary brain injury; however, effective early biomarkers for vasospasm are currently lacking. At present, clinicians have little choice but to monitor and then react once vasospasm or a delayed ischemic deficit becomes evident, underscoring the need for predictive biomarkers [20]. In the era of precision medicine, identifying molecular indicators of vasospasm risk could enable proactive interventions and tailored patient management [17]. Numerous studies have highlighted the potential of miRNAs as biomarkers for complications following aSAH. Most have found that changes in miRNA expression correlate with aSAH occurrence and its acute complications and that these changes can be detected in extracellular fluids [14]. Nonetheless, no miRNA has yet been validated for clinical use to reliably distinguish which aSAH patients will develop vasospasm (one of the most critical complications) and therefore might benefit from early aggressive therapy, versus those who will not. Given the tissue−specific nature of miRNAs, it is important to focus on miRNAs in cerebrospinal fluid (CSF)—which reflects brain−derived miRNAs—when studying central complications like vasospasm. Indeed, CSF miRNAs are considered potentially better candidates for predicting vasospasm and related outcomes than peripheral miRNAs [9]. To our knowledge, only a limited number of prior studies have examined CSF miRNAs in aSAH in the context of vasospasm or DCI, underscoring that this is an emerging area of research. Our study contributes to this area by profiling 20 candidate miRNAs in CSF and identifying those associated with vasospasm development and severity.

In this study, we assessed CSF samples from aSAH patients on days 1 and 5 post−hemorrhage, comparing those who developed vasospasm (VSP+) to those who did not (VSP−), as well as to non−SAH controls. We identified several miRNAs with significant differential expression linked to vasospasm. Notably, on the first day after SAH, miR−221−3p, miR−9−3p, and miR−183−5p showed statistically significant differences between the VSP+ and VSP− groups, even before vasospasm clinically manifested. In VSP+ cases, miR−221−3p was substantially lower (approximately an 8−fold decrease relative to VSP−), whereas miR−9−3p (~13−fold higher) and miR−183−5p (~2.7−fold higher) were elevated. These early alterations were strong enough to distinguish between the two patient groups, with ROC analyses indicating high specificity, sensitivity, and AUC values (miR−9−3p, AUC ~0.92; miR−221−3p, AUC ~0.81; miR−183−5p, AUC ~0.76). Thus, just one day after SAH, these circulating miRNAs demonstrated strong potential as biomarkers for predicting which patients will develop vasospasm.

Most importantly, at 5 days post−SAH (approximately the typical vasospasm window) there were no significant differences between the groups in the given miRNAs, indicating that the discriminatory window is present in the very early stage. In contrast, focusing on the comparison of VSP+ patients to non−SAH controls, we observed a larger number of miRNAs (five on day 1) that were different, indicative of the overall injury response to SAH with vasospasm. MiR−221−3p and miR−183−5p were dramatically upregulated in VSP+ vs. controls on day 1, and miR−126, miR−29a−3p, and miR−27b−3p were downregulated with persistence of markedly low values particularly for miR−126 on day 5. These results are also partially consistent with the previous findings that reported alterations in these miRNAs in different brain injuries. For instance, miR−126 is abundant in endothelial cells and involved in vascular homeostasis, so its under−expression after SAH could indicate the endothelial impairment and blood–brain barrier breakdown that mediate vasospasm physiopathology [21]. The sustained suppression of miR−126 in our vasospasm patients aligns with its proposed role in maintaining cerebrovascular stability. Our study also found that several miRNAs correlated with clinical severity and outcomes among vasospasm patients. Notably, miR−17−5p, miR−183−5p, and miR−126 showed a significant positive correlation in expression from day 1 to day 5 in VSP+ patients (i.e., their levels rose or remained low in tandem over time), hinting at persistent dysregulation through the vasospasm period. By day 5, patients with poor neurological status (higher Hunt–Hess, higher WFNS, and worse mRS outcomes) had significantly higher CSF levels of miR−183−5p (across all three severity scales) and miR−17−5p (in Fisher grade and mRS comparisons) compared with those with milder presentations. In particular, miR−183−5p stood out: its expression was significantly higher on day 5 in VSP+ patients who had poor outcomes (mRS 3–6) compared with those with good recovery (mRS 1–2). This suggests that miR−183−5p might be associated not only with vasospasm occurrence but also with the extent of resulting brain injury and recovery. Furthermore, miR−21−5p, miR−221−3p, and miR−24 were all significantly upregulated in VSP+ patients with more severe clinical presentations and poorer prognoses, which is particularly noteworthy. These miRNAs have been implicated in vascular inflammation and remodeling (as discussed below), and their elevated levels in more severe cases underscore their potential relevance as prognostic markers.

Some of the miRNAs identified in this study have been linked in the literature to SAH complications. For instance, Su et al. (2015) reported that miR−132−3p levels differed in blood samples of patients with delayed cerebral infarction (DCI) versus those without [22]. Bache et al. (2017) found significantly elevated miR−21−5p and miR−221−3p in the CSF of patients who developed DCI and noted involvement of miR−221−3p, miR−132−3p, and miR−19b−3p in those who had vasospasm [14]. Stylli et al. (2017) observed that CSF levels of miR−27a−3p differed between aSAH patients with and without delayed cerebral vasospasm [10]. In a blood−based study, Lopes et al. (2018)—using next−generation sequencing—found significant differences in miRNA profiles between aSAH patients and controls, but not between those with vs. without vasospasm, possibly due to later sampling times (days 7 and 10 in that study) when differences had evened out [23]. More recently, Wang et al. (2021) examined 47 miRNAs in CSF and plasma on post−bleed days 3 and 7 and reported that certain miRNAs had significantly different patterns in patients with DCI, as early as day 3 [9]. They suggested that these could predict DCI onset before clinical signs. Similarly, Bache et al. (2020) analyzed CSF miRNA profiles and found that increased miR−9−3p in CSF was associated with worse 3−month functional outcomes (mRS 3–6) [24]. This is intriguing, as our study also found miR−9−3p to be elevated in vasospasm patients (vs. non−vasospasm) and it was one of the strongest early markers; however, in our cohort, miR−9−3p itself did not correlate with long−term outcome, perhaps because all our VSP+ patients had significant brain injury. In general, our findings align with these prior studies in highlighting specific miRNAs (e.g., miR−21, miR−24, miR−132, miR−221, and miR−9) as relevant in SAH and its complications. Differences between studies likely stem from variations in patient cohorts, sample type (CSF vs. blood), timing of sample collection, and technical methodologies. These factors make direct comparisons challenging, emphasizing the need for more standardized research in this field.

Among the miRNAs we studied, miR−24 emerged as particularly interesting. Recent reports indicate that miR−24 is involved in various diseases through effects on inflammatory signaling pathways. Several studies suggest that upregulation of miR−24 can contribute to SAH−induced cerebrovascular complications by inhibiting endothelial nitric oxide synthase (NOS3, i.e., eNOS) [25,26]. eNOS continuously produces nitric oxide, which is essential for maintaining basal vascular tone; after SAH, reductions in eNOS activity can upset cerebral vascular tone and promote vasospasm [25]. Additionally, miR−24 can target heme oxygenase−1 (HMOX1). Following SAH, the degradation of heme from extravasated blood by heme oxygenase enzymes (especially inducible HMOX1) is neuroprotective; loss of HMOX1 exacerbates inflammation and oxidative injury. Notably, miR−24 has been shown to negatively regulate both NOS3 and HMOX1 expression. In a rat SAH model, increased miR−24 was associated with decreased NOS3/HMOX1 levels and worse vasospasm, whereas experimentally inhibiting miR−24 led to increased HMOX1 and a partial recovery from vasospasm−induced brain injury [27]. In our study, we observed a significant increase in miR−24 expression in VSP+ patients, particularly by day 5 after SAH. This timing coincides with the typical peak of vasospasm and secondary injury, and our data showed that miR−24 levels were higher in patients with severe clinical grades (Hunt–Hess 3–5) and poor outcomes (mRS 3–6) compared with those with milder presentations. These observations support the notion that miR−24 is a candidate molecular marker for vasospasm development and a prognostic indicator (higher miR−24 may identify patients at greater risk of vasospasm−related deficits, who might benefit from more aggressive monitoring or experimental therapies). It is noteworthy that miR−24 levels have been found to be elevated in aSAH patients with angiographic vasospasm compared with those without [26] and that downregulating miR−24 can mitigate vasospasm injury in experimental models [27]. To our knowledge, our study is the first to demonstrate an association between increased CSF miR−24 and poor patient outcomes after aSAH with vasospasm, highlighting miR−24’s potential as a prognostic biomarker.

Turning to miR−221−3p, this miRNA has been implicated by multiple studies in cerebral vasospasm after SAH. MiR−221−3p’s functions involve modulating vascular smooth muscle cell (VSMC) behavior, inflammatory responses, and vascular remodeling. It is known to promote VSMC proliferation and shift VSMCs to a less contractile, more proliferative phenotype by targeting cell cycle regulators like p27^Kip1 and p57^Kip2. This leads to neointimal hyperplasia and a narrowed vessel lumen, which can contribute to vasospasm. MiR−221 (and the closely related miR−222) have also been shown to increase PDGF−induced VSMC migration and phenotypic modulation [28]. MiR−221−3p can also affect endothelial function and inflammation; elevated miR−221−3p may impair angiogenesis and promote an inflammatory microenvironment by influencing endothelial cell proliferation and migration and possibly cytokine production and leukocyte adhesion [29]. Clinically, CSF levels of miR−221−3p have been reported to be higher in SAH patients with angiographic vasospasm [14]. A recent meta−analysis by Li et al. (2023) identified miR−221−3p as a potential biomarker for predicting neurological outcomes after SAH. Patients with good outcomes had significantly lower miR−221−3p levels than those with poor outcomes [30]. Our findings are in line with these reports. We found that high miR−221−3p expression was associated with vasospasm risk (it was elevated in VSP+ vs. controls and was a strong predictor in ROC analysis on day 1). Yet, paradoxically, when comparing VSP+ vs. VSP− patients directly, we observed that miR−221−3p was lower in the VSP+ group on day 1. This apparent contradiction might be explained by timing and the dual role of miR−221−3p. It rises after SAH in general, but perhaps an early relative deficiency or delayed rise in VSP+ patients could signal a dysregulated response that predisposes to vasospasm. By day 5, we observed that higher miR−221−3p levels were indeed associated with worse clinical grades among VSP+ patients, and, importantly, the highest miR−221−3p levels were found in VSP+ patients with poor outcomes across all severity scales (Hunt–Hess, WFNS, mRS). This aligns with the notion that a sustained elevation of miR−221−3p is detrimental [30]. In short, our study confirms miR−221−3p as both a predictive and prognostic biomarker in aSAH. Its early aberrant levels herald vasospasm, and persistently high levels are associated with worse neurological outcomes.

The role of miR−183−5p in vasospasm is less established, but our data suggest that it may be significant. MiR−183−5p has been studied in other vascular and neurological contexts. In vascular diseases like atherosclerosis, miR−183−5p promotes VSMC proliferation and migration, contributing to vascular remodeling and stenosis [31]. This mechanism could be relevant to vasospasm, where VSMC contraction and proliferation play a part in vessel narrowing. Additionally, miR−183−5p is involved in inflammatory responses and oxidative stress. In intracerebral hemorrhage (ICH) models, miR−183−5p was found to be decreased, and its overexpression alleviated early brain injury by reducing oxidative stress and neuroinflammation—partly via inhibiting HO−1 [32]. Direct evidence of miR−183−5p in vasospasm per se has been lacking; our study provides new insight by showing that miR−183−5p was significantly elevated in vasospasm patients vs. non−vasospasm early on, and that a greater rise in miR−183−5p over time was associated with poorer neurological outcomes. We did not find prior literature linking miR−183−5p to vasospasm prognosis, but our data suggest that miR−183−5p could contribute to vasospasm pathophysiology through its effects on VSMCs, inflammation, and angiogenesis. Notably, its upregulation may not be a protective change—rather, higher miR−183−5p in CSF might reflect more severe vascular injury or a maladaptive response. Given the limited prior evidence, further research is needed to clarify miR−183−5p’s role. Nonetheless, our findings indicate that it has value in differentiating vasospasm risk early and is worth investigating as part of a biomarker panel. It is conceivable that miR−183−5p, alongside miR−221−3p and miR−9−3p, forms a signature of acute vascular injury from SAH that predisposes to vasospasm.

MiR−21 is one of the most extensively studied miRNAs in vascular biology and has been implicated in vasospasm after SAH. MiR−21’s functions are multifaceted, including regulation of inflammation, VSMC proliferation, apoptosis inhibition, and endothelial protection. It is generally upregulated after SAH and other brain injuries [14]. MiR−21 targets multiple pro−apoptotic and pro−inflammatory genes (e.g., PDCD4, PTEN, FasL, and IL6R), thereby attenuating cell death and inflammation [33]. In the context of vasospasm, miR−21 may act as a double−edged sword. On one hand, it can promote VSMC proliferation and phenotypic modulation (potentially contributing to vessel wall thickening and vasoconstriction). On the other hand, it has protective effects on endothelial cells and neurons, mitigating apoptosis and blood–brain barrier breakdown [28]. In our study, miR−21−5p was not among the earliest differentiators of vasospasm vs. no vasospasm on day 1 (its levels did not significantly differ then), but by day 5 we found that miR−21−5p was significantly higher in VSP+ patients with severe grades and poor outcomes compared with those with better clinical status. This suggests that miR−21−5p elevation is more a marker of ongoing injury severity rather than a cause of vasospasm onset. Still, it showed discriminative ability between VSP+ and VSP− on day 5 (ROC AUC ~0.81 for certain severity comparisons). Elevated CSF miR−21−5p has been noted in previous studies to associate with poorer outcomes or more severe SAH complications. For instance, a relative increase in miR−21 was observed in the CSF of patients who went on to develop late cerebral ischemia (versus those who did not) [34]. Our results concur: miR−21−5p levels were higher in patients with worse neurological outcomes at 3 months, versus those with good outcomes. This miRNA might be useful as part of a prognostic biomarker panel.

Finally, it is essential to recognize the limitations to our study. One limitation of this study is the relatively small number of control samples, which is primarily due to the ethical and procedural constraints of obtaining CSF from neurologically healthy individuals. Despite the low inter−individual variability observed in the control group, future studies with larger and demographically matched control cohorts will be necessary to enhance the statistical power and generalizability of the findings. The sample size, although moderate for a specialized study, can be considered small—especially if stratifying patients according to clinical grades—which compromises statistical power and external validity. Although our results are encouraging, validation in larger independent cohorts is needed. The observational design can demonstrate association, not causation. We did not confront this lack of biological support with hard, mechanistic studies testing direct targets of these miRNAs in humans (for example, measuring NOS3 or HMOX1 protein levels in parallel with the changes in miR−24 expression), which would have strongly reinforced the biological connection. Second, although we obtained samples at two time points (day 1 and day 5), vasospasm can occur at different time points; some patients may develop vasospasm earlier or later than our sampling window. Another limitation of this study is the absence of exogenous spike−in and multiple controls (e.g., cel−miR−39), which could have further improved normalization accuracy and quality control. Although U6 showed stable expression across all samples, incorporating exogenous controls in future studies will allow for more effective correction of technical variability and enhance the reproducibility of results. A shorter sampling interval could detect more dynamic changes. We were also limited in our studies to the expression of a pre−determined panel of 20 miRNAs, and so we cannot exclude the potential involvement of other unexamined miRNAs in vasospasm. Further studies using unbiased high−throughput sequencing or arrays will probably identify more genes. However, despite these limitations, our study has yielded innovative preliminary evidence suggesting that CSF miRNAs may represent early markers of vasospasm and a significant measure of disease severity. It paves the way for more comprehensive studies with the particular miRNAs identified as worthy of further investigation.

## 5. Conclusions

To conclude, this study identified distinct CSF miRNA expression alterations that precede and accompany vasospasm after aSAH. In this study, differences in the expression of CSF miRNAs were detected between the pre−vasospasm state and the vasospasm state after aSAH. Very high discrimination power (at 1 day post−hemorrhage) for predicting vasospasm in the first place by miR−221−3p, miR−9−3p, and miR−183−5p was found. Increased levels of miR−24 and miR−21−5p correlated to severe clinical symptoms and poor outcome, indicating possible biomarker value. These miRNAs—in particular in combination—are promising targets for an early diagnostic and risk stratification panel. Our results also underscore the pathogenetic importance of miRNA pathways associated with inflammation and endothelial dysfunction in vasospasm pathogenesis. While these are promising results, they are only preliminary. Extensive validation is warranted to confirm clinical utility, to determine an appropriate cut−off value, and to form a reliable multi−marker miRNA model predictive of future LV adaptation. Moreover, miRNA biomarkers, with further research, could be a potent weapon for enhancing the diagnosis, prognosis, and personalized treatment of vasospasm in aSAH patients. 

## Figures and Tables

**Table 1:** Demographic characteristics of the study population (high clinical grades are included in the table).

	Control (n = 8)	Vasospasm + (n = 20)	Vasospasm − (n = 20)	*p*-Value
**Age**	57.75 ± 14.17	57.95 ± 12.02	57.12 ± 9.73	0.988
**Sex (female)**	4 (%50)	9 (%45)	10 (%50)	0.943
**Hypertension**	NDA	7 (%35)	6 (%30)	0.736
**Fisher grade (4)**	−	11 (%55)	12 (%60)	0.749
**Hunt–Hess grade (3–5)**	−	8 (%40)	6 (%30)	0.507
**WFNS grade (3–5)**	−	6 (%30)	4 (%20)	0.465
**Modified Rankin Scale (3–6)**	−	13 (%65)	8 (%40)	0.113

NDA, no data available; WFNS, World Federation of Neurosurgical Societies.

**Table 2:** Circulating miRNA levels examined in CSF samples of vasospasm−positive patients and the control group.

Vasospasm Positive and Control Group (1st Day After SAH)				Vasospasm−Positive and Control Group (5th Day After SAH)			
miRNA	Fold Changes	*p* Value	Regulation	miRNA	Fold Changes	*p* Value	Regulation
miR−221−3p	4.04	0.014 *	Upregulation	miR−126	0.18	0.002 *	Downregulation
miR−183−5p	3.05	0.009 *	Upregulation				
miR−126	0.19	0.006 *	Downregulation				
miR−29a	0.17	0.021 *	Downregulation				
miR−27b−3p	0.46	0.028 *	Downregulation				

*, <0.05 *p* value is statistically significant; SAH, subarachnoid hemorrhage.

**Table 3:** ROC and AUC values of circulating miRNAs examined in CSF samples of vasospasm−positive−patients and the control group.

Vasospasm−Positive and Control Group (1st Day After SAH)						Vasospasm−Positive and Control Group (5th Day After SAH)					
miRNA	Sensitivity	Specificity	AUC (%95 CI)	Cut−Off	*p* Value	miRNA	Sensitivity	Specificity	AUC (%95 CI)	Cut−Off	*p* Value
miR−221−3p	100.00	87.50	0.975	≤3.45	0.0001 *	miR−126	100.00	7.50	**0.981**	>3.05	<0.0001 *
(0.833–1.000)						(0.843–1.000)					
miR−183−5p	80.00	62.50	0.744	≤0.72	0.0186 *						
(0.545–0.889)											
miR−126	95.00	100.00	0.975	>3.66	< 0.0001 *						
(0.833–1.000)											
miR−29a-3p	45.00	87.50	0.625	>9	0.262						
(0.423–0.799)											
miR−27b−3p	80.00	100.0	0.963	>9.98	<0.0001 *						
(0.814–0.999)											

*, <0.05 *p*-value is statistically significant; CI, confidence interval; AUC, area under the curve.

**Table 4:** Circulating miRNA levels examined in CSF samples of vasospasm−negative patients and the control group.

Vasospasm−Negative and Control Group (1st Day After SAH)						Vasospasm−Negative and Control Group (5 Days After SAH)					
miRNA	Sensitivity	Specificity	AUC (%95 CI)	Cut−Off	*p* Value	miRNA	Sensitivity	Specificity	AUC (%95 CI)	Cut−Off	*p* Value
miR−221−3p	95.00	100.00	0.994	≤2.93	<0.0001 *	miR−29a	55.00	87.00	**0.650**	>9	0.151
(0.865–1.000)						(0.448–0.819)					
miR−9−3p	70.00	87.50	0.781	>−0.42	0.0018 *						
(0.585–0.914)											
miR−29a	50.00	87.50	0.619	>9	0.284						
(0.417–0.794)											

*, <0.05 *p*-value is statistically significant; CI, confidence interval; AUC, area under the curve.

**Table 5:** Comparison of miRNA expression profiles in samples of patients with and without vasospasm.

miRNA	Sensitive	Specificity	AUC (%95 CI)	Cut−Off	*p* Value
miR−221−3p	95.00	75.00	0.812 (0.658–0.918)	>−0.89	<0.0001 *
miR−9−3p	80.00	85.00	0.915 (0.783–0.980)	≤−2.05	<0.0001 *
miR−183−5p	85.00	65.00	0.756 (0.595–0.878)	≤0.86	0.001 *

*, <0.05 *p*−value is statistically significant; CI, confidence interval; AUC, area under the curve. Comparison of miRNA expression profiles with clinical features in cases developing vasospasm (Fisher score, Hunt–Hess score, WFNS score, and modified Rankin scale). In VSP+ cases, the Fisher score, Hunt–Hess score, WFNS score, and modified Rankin scale were used in clinical evaluation. The patients were divided into two groups according to mild and severe clinical findings, and miRNA expression levels were compared between the two groups.

**Table 6:** ROC and AUC values of circulating miRNAs analyzed between Fisher grade groups in SAH Day 5 samples of cases with vasospasm.

miRNA	Sensitivity	Specificity	AUC (%95 CI)	Cut−Off	*p* Value
miR−17−5p	63.64	100.00	0.843 (0.613−0.965)	≤10.31	0.0003 *
miR−21−5p	72.73	88.89	0.808 (0.572−0.947)	≤−0.12	0.0039 *
miR−221−3p	72.73	88.89	0.813 (0.578−0.950)	≤−0.39	0.002 *
miR−24 *	63.64	88.89	0.768 (0.528−0.924)	≤2.42	0.020 *

*, <0.05 *p*−value is statistically significant; CI, confidence interval; AUC, area under the curve.

**Table 7:** ROC and AUC values of circulating miRNAs analyzed among neurologic clinical features in samples of cases with vasospasm.

	miRNA	Sensitivity	Specificity	AUC (%95 CI)	Cut−Off	*p* Value
Hunt–Hess 3–5 vs. 1–2	miR−148b−3p	75.00	91.67	0.833 (0.601–0.960)	≤11.46	0.0025 *
	miR−21−5p	87.50	83.33	0.813 (0.577–0.949)	≤1.35	0.0114 *
	miR−17−5p	87.50	66.67	0.786 (0.548–0.935)	≤12.5	0.0073 *
	miR−221−3p	100.00	66.67	0.807 (0.572–0.947)	≤1.2	0.0027 *
	miR−183−5p	87.50	83.33	0.865 (0.639–0.974)	≤−0.22	<0.001 *
	miR−497	87.50	75.00	0.802 (0.566–0.944)	≤3.04	0.0035 *
	miR−24	100.00	66.67	0.813 (0.577–0.949)	≤3.24	0.0021 *
	miR−143−3p	100.00	66.67	0.875 (0.651–0.979)	≤3.79	<0.001 *
	miR−145−5p	100.00	66.67	0.813 (0.577–0.949)	≤9.34	0.0021 *
	miR−27b−3p	75.00	83.33	0.823 (0.589–0.955)	≤8.95	0.0013 *
	miR−502−5p	87.50	58.33	0.771 (0.531–0.926)	≤10.68	0.0116 *
WFNS 3–5 vs. 1–2	miR−221−3p	100.00	64.29	0.815 (0.581–0.951)	≤0.77	0.0014 *
	miR−183−5p	83.33	78.57	0.845 (0.615–0.966)	≤−0.29	0.0004 *
	miR−143−3p	83.33	78.57	0.857 (0.630–0.971)	≤1.82	0.001 *
	miR−145−5p	66.67	92.86	0.821 (0.588–0.954)	≤5.67	0.0016 *
	miR−27b−3p	83.33	78.57	0.821 (0.588–0.954)	≤8.95	0.0030 *

*, <0.05 *p*−value is statistically significant; CI, confidence interval; AUC, area under the curve.

## Data Availability

The data used in this study will be shared only upon request due to ethical restrictions.

## Abbreviations

The following abbreviations are used in this manuscript:

aSAH	aneurysmal subarachnoid hemorrhage
CSF	cerebrospinal fluid
miRNAs	micro ribonucleic acids
PCR	polymerase chain reaction
VSP+	vasospasm positive
VSP−	vasospasm negative
WFNS	World Federation of Neurosurgical Societies
HH	Hunt–Hess score
GCS	Glasgow Coma Scale
mRS	Modified Rankin Scale
ROC	receiver operating characteristic
AUC	area under the curve
CI	confidence interval

## References

[1] Tawk R.G., Hasan T.F., D’Souza C.E., Peel J.B., Freeman W.D. (2021). Diagnosis and Treatment of Unruptured Intracranial Aneurysms and Aneurysmal Subarachnoid Hemorrhage. Mayo Clin. Proc..

[2] Seule M., Oswald D., Muroi C., Brandi G., Keller E. (2020). Outcome, Return to Work and Health-Related Costs After Aneurysmal Subarachnoid Hemorrhage. Neurocrit. Care.

[3] Chung D.Y., Abdalkader M., Nguyen T.N. (2021). Aneurysmal Subarachnoid Hemorrhage. Neurol. Clin..

[4] Janjua N., Mayer S.A. (2003). Cerebral vasospasm after subarachnoid hemorrhage. Curr. Opin. Crit. Care.

[5] Muraoka S., Izumi T., Nishihori M., Goto S., Takeuchi I., Saito R. (2025). Emerging Advances in the Management of Delayed Cerebral Ischemia After Aneurysmal Subarachnoid Hemorrhage: A Narrative Review. J. Clin. Med..

[6] Dodd W.S., Laurent D., Dumont A.S., Hasan D.M., Jabbour P.M., Starke R.M., Hosaka K., Polifka A.J., Hoh B.L., Chalouhi N. (2021). Pathophysiology of Delayed Cerebral Ischemia After Subarachnoid Hemorrhage: A Review. J. Am. Heart Assoc..

[7] Huntzinger E., Izaurralde E. (2011). Gene silencing by microRNAs: Contributions of translational repression and mRNA decay. Nat. Rev. Genet..

[8] Iwakawa H.O., Tomari Y. (2015). The Functions of MicroRNAs: mRNA Decay and Translational Repression. Trends Cell Biol..

[9] Wang W.X., Springer J.E., Xie K., Fardo D.W., Hatton K.W. (2021). A Highly Predictive MicroRNA Panel for Determining Delayed Cerebral Vasospasm Risk Following Aneurysmal Subarachnoid Hemorrhage. Front. Mol. Biosci..

[10] Stylli S.S., Adamides A.A., Koldej R.M., Luwor R.B., Ritchie D.S., Ziogas J., Kaye A.H. (2017). miRNA expression profiling of cerebrospinal fluid in patients with aneurysmal subarachnoid hemorrhage. J. Neurosurg..

[11] Mraz M., Malinova K., Mayer J., Pospisilova S. (2009). MicroRNA isolation and stability in stored RNA samples. Biochem. Biophys. Res. Commun..

[12] Etheridge A., Lee I., Hood L., Galas D., Wang K. (2011). Extracellular microRNA: A new source of biomarkers. Mutat. Res..

[13] Segherlou Z.H., Saldarriaga L., Azizi E., Vo K.A., Reddy R., Siyanaki M.R.H., Lucke-Wold B. (2023). MicroRNAs’ Role in Diagnosis and Treatment of Subarachnoid Hemorrhage. Diseases.

[14] Bache S., Rasmussen R., Rossing M., Laigaard F.P., Nielsen F.C., Møller K. (2017). MicroRNA Changes in Cerebrospinal Fluid After Subarachnoid Hemorrhage. Stroke.

[15] Wang L., Luo Y.C., Wang H., Zou Y.B., Yao H.Q., Ullah S., Li Z.Q. (2020). Azure-winged magpies fail to understand the principle of mirror imaging. Behav. Process..

[16] Ban E., Song E.J. (2022). Considerations and Suggestions for the Reliable Analysis of miRNA in Plasma Using qRT-PCR. Genes.

[17] Przybycien-Szymanska M.M., Ashley W.W. (2015). Biomarker Discovery in Cerebral Vasospasm after Aneurysmal Subarachnoid Hemorrhage. J. Stroke Cerebrovasc Dis..

[18] Chou S.H. (2018). Inflammation, Cerebral Vasospasm, and Brain Injury in Subarachnoid Hemorrhage-A Shifting Paradigm and a New Beginning. Crit. Care Med..

[19] Carr K.R., Zuckerman S.L., Mocco J. (2013). Inflammation, cerebral vasospasm, and evolving theories of delayed cerebral ischemia. Neurol. Res. Int..

[20] Mocco J., Zacharia B.E., Komotar R.J., Connolly E.S. (2006). A review of current and future medical therapies for cerebral vasospasm following aneurysmal subarachnoid hemorrhage. Neurosurg. Focus.

[21] Fu X., Niu T., Li X. (2019). MicroRNA-126-3p Attenuates Intracerebral Hemorrhage-Induced Blood-Brain Barrier Disruption by Regulating VCAM-1 Expression. Front. Neurosci..

[22] Su X.W., Chan A.H., Lu G., Lin M., Sze J., Zhou J.Y., Poon W.S., Liu Q., Zheng V.Z., Wong G.K. (2015). Circulating microRNA 132-3p and 324-3p Profiles in Patients after Acute Aneurysmal Subarachnoid Hemorrhage. PLoS ONE.

[23] Lopes K.P., Vinasco-Sandoval T., Vialle R.A., Paschoal F.M., Bastos V.A.P.A., Bor-Seng-Shu E., Teixeira M.J., Yamada E.S., Pinto P., Vidal A.F. (2018). Global miRNA expression profile reveals novel molecular players in aneurysmal subarachnoid haemorrhage. Sci. Rep..

[24] Bache S., Rasmussen R., Wolcott Z., Rossing M., Møgelvang R., Tolnai D., Hassager C., Forman J.L., Køber L., Nielsen F.C. (2020). Elevated miR-9 in Cerebrospinal Fluid Is Associated with Poor Functional Outcome After Subarachnoid Hemorrhage. Transl. Stroke Res..

[25] Li H.T., Wang J., Li S.F., Cheng L., Tang W.Z., Feng Y.G. (2018). Upregulation of microRNA-24 causes vasospasm following subarachnoid hemorrhage by suppressing the expression of endothelial nitric oxide synthase. Mol. Med. Rep..

[26] Pulcrano-Nicolas A.S., Proust C., Clarençon F., Jacquens A., Perret C., Roux M., Shotar E., Thibord F., Puybasset L., Garnier S. (2018). Whole-Blood miRNA Sequencing Profiling for Vasospasm in Patients With Aneurysmal Subarachnoid Hemorrhage. Stroke.

[27] Deng X., Liang C., Qian L., Zhang Q. (2021). miR-24 targets HMOX1 to regulate inflammation and neurofunction in rats with cerebral vasospasm after subarachnoid hemorrhage. Am. J. Transl. Res..

[28] Yu X., Li Z. (2014). MicroRNAs regulate vascular smooth muscle cell functions in atherosclerosis (Review). Int. J. Mol. Med..

[29] Li Y., Yan C., Fan J., Hou Z., Han Y. (2021). MiR-221-3p targets Hif-1α to inhibit angiogenesis in heart failure. Lab. Investig..

[30] Ten Brinck M.F.M., Shimanskaya V.E., Aquarius R., Bartels R.H.M.A., Meijer F.J.A., Koopmans P.C., de Jong G., Wakhloo A.K., de Vries J., Boogaarts H.D. (2022). Outcomes after Flow Diverter Treatment in Subarachnoid Hemorrhage: A Meta-Analysis and Development of a Clinical Prediction Model (OUTFLOW). Brain Sci..

[31] Lv D., Guo Y., Zhang L., Li X., Li G. (2023). Circulating miR-183-5p levels are positively associated with the presence and severity of coronary artery disease. Front. Cardiovasc. Med..

[32] Zhang J., Li A., Gu R., Tong Y., Cheng J. (2023). Role and regulatory mechanism of microRNA mediated neuroinflammation in neuronal system diseases. Front. Immunol..

[33] Kousar K., Ahmad T., Abduh M.S., Kanwal B., Shah S.S., Naseer F., Anjum S. (2022). miRNAs in Regulation of Tumor Microenvironment, Chemotherapy Resistance, Immunotherapy Modulation and miRNA Therapeutics in Cancer. Int. J. Mol. Sci..

[34] Makowska M., Smolarz B., Romanowicz H. (2022). microRNAs in Subarachnoid Hemorrhage (Review of Literature). J. Clin. Med..

